# In vivo mapping of infant brain microstructure with neurite orientation dispersion and density imaging

**DOI:** 10.1007/s00429-025-03007-2

**Published:** 2025-09-25

**Authors:** Yanbin Niu, M. Catalina Camacho, Kurt G. Schilling, Kathryn L. Humphreys

**Affiliations:** 1 Department of Psychology and Human Development, Peabody College, Vanderbilt University, 230 Appleton Place, #552, Nashville, TN 37203 USA; 2 Department of Psychiatry, Washington University in St. Louis, St. Louis, MO USA; 3 Department of Radiology and Radiological sciences (VUMC), Vanderbilt University Medical Center, Nashville, TN USA

**Keywords:** NODDI, Neurodevelopment, Microstructure, Infancy

## Abstract

Diffusion magnetic resonance imaging (dMRI) is a non-invasive neuroimaging technique that measures the displacement of water molecules in tissue over time. Due to its sensitivity to micron-scale water movement, which is influenced by cellular structures like membranes, axons, and myelin, dMRI is a unique method for probing tissue microstructure. Among dMRI analysis approaches, neurite orientation dispersion and density imaging (NODDI) is a biophysical modeling technique that enables the characterization of cytoarchitectural and myeloarchitectural features in the brain. The early postnatal period is characterized by rapid and dynamic biological processes such as axonal growth, dendritic arborization, and synaptogenesis—changes that alter the microstructural environment in ways that are detectable by NODDI. Thus, NODDI presents a promising approach for characterizing early brain development, offering biologically specific markers of tissue organization that are responsive to these maturational events. This review presents emerging literature on NODDI applications during early infancy, demonstrating its utility in mapping normative developmental trajectories, investigating alterations in preterm populations, and linking microstructural properties to environmental influences and emerging behavioral outcomes. While current literature offers initial insights into early microstructural development patterns, NODDI applications in infancy remain limited, and existing studies are constrained by small sample sizes, limited age coverage, and lack of longitudinal data. Nonetheless, initial evidence suggests that NODDI can complement conventional diffusion metrics and may provide novel insights into early neural maturation and plasticity. Continued application and methodological refinement of NODDI in infancy may help delineate sensitive periods of brain development and improve the interpretation of emerging neurobehavioral phenotypes.

## Introduction

Diffusion magnetic resonance imaging (dMRI) is a non-invasive neuroimaging technique that measures the displacement of water molecules in tissue over time. Due to its sensitivity to micron-scale water movement, dMRI is a unique method for probing tissue microstructure (Beaulieu 2002). By providing insights into the underlying architecture of neural tissues in vivo, dMRI has become an invaluable tool for studying the developing brain (DiPiero et al. 2023b). Diffusion tensor imaging (DTI), a widely used dMRI analysis technique, models water diffusion as a tensor, offering metrics that describe the anisotropy or isotropy of water movement within tissues (Basser et al. 1994), and has long been an important tool in clinical diagnosis and research (Pasternak et al. 2018). More recent approaches have moved beyond the tensor model to incorporate biophysical models that allow for more advanced and specific characterization of tissue microstructure. Biophysical models aim for specificity by parametrizing the dMRI signal as a function of biophysically meaningful parameters (Jelescu et al. 2020). One such biophysical model is neurite orientation dispersion and density imaging (NODDI) (Zhang et al. 2012), which models the brain’s microstructure by separating water diffusion into different compartments, such as intracellular and extracellular spaces.

During early infancy, brain development is marked by rapid and dynamic biological processes such as axonal growth, dendritic arborization, synaptogenesis, and early myelination. NODDI offers a window into these cellular-level developmental processes by providing compartment-specific metrics—such as neurite density and orientation dispersion—that reflect features of cytoarchitecture and neurite geometry. In addition to its biological specificity, NODDI is also technically feasible for use in infants, making it a promising tool for studying early brain development. NODDI requires only two b-value shells in addition to b = 0 images, and does not demand ultra-high b-values (e.g., greater than 2500 s/mm^2^) (Zhang et al. 2012), making it compatible with standard clinical MRI scanners without specialized hardware. Scan durations can be as short as 4 min (Kunz et al. 2014), a duration well-suited to natural sleep scanning in infants. Moreover, because it requires no contrast agents or sedation, and has demonstrated high reliability across repeated measurements (Tariq et al. 2012), NODDI is well-suited for longitudinal research, allowing researchers to safely and non-invasively track dynamic microstructural changes in vivo across the first months of life—a period marked by rapid and foundational brain development. In this review, we begin by outlining the technical foundations of NODDI, including its multi-compartment modeling framework, biophysical basis, and key limitations. We then provide an overview of early microstructural development, followed by applications of NODDI in mapping microstructural development during infancy. Next, we review microstructural development in preterm infants and links between early brain microstructure, behavioral development, environmental factors, and genetics. We conclude by discussing the relevance of NODDI to developmental psychology and outlining future research directions.

## Neurite orientation dispersion and density imaging (NODDI)

### Multi-compartment modeling in NODDI

NODDI is a diffusion MRI technique based on multi-compartment modeling (Zhang et al. 2012). Multi-compartment models interpret the signals measured in each voxel as the sum of contributions from distinct compartments within that voxel, such as intracellular and extracellular spaces. This approach confers a fundamental advantage over the standard DTI, which models water diffusion as a single tensor and only provides a composite view of the multifaceted contributions that may exist in a voxel (Zhang et al. 2012). A change to the DTI metric fractional anisotropy, for example, may be caused by a host of underlying changes to the contributing compartments. In contrast, NODDI aims to disentangle the contribution from each compartment, thereby enabling their individualized characterization. By accounting for multiple compartments, NODDI provides a more granular and biologically meaningful representation of brain tissue microstructure.

**Fig. 1:**
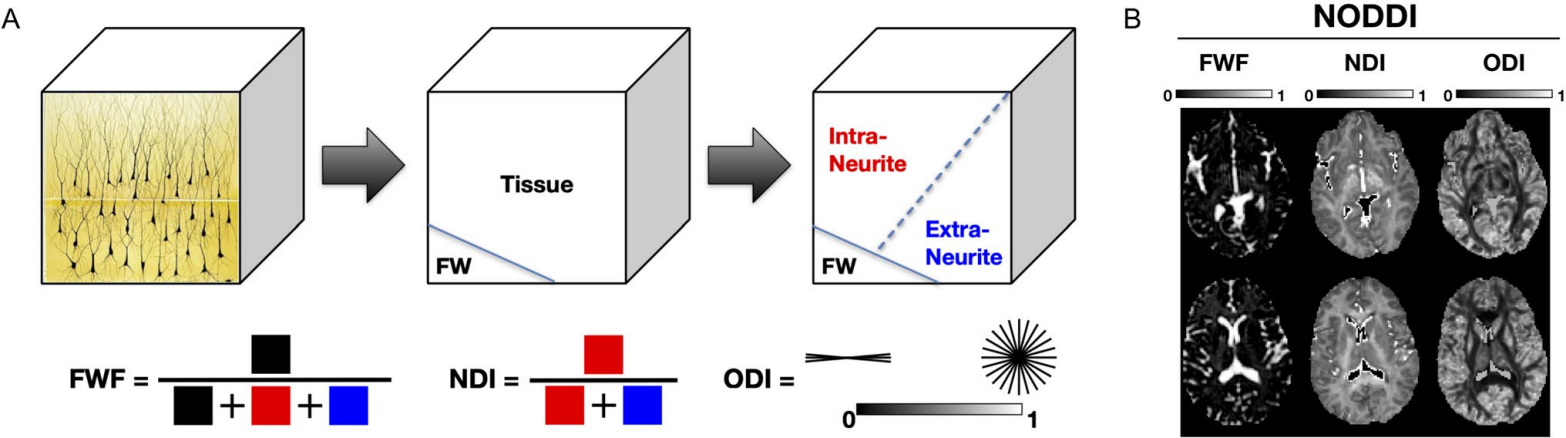
NODDI model components and representative maps of NODDI parameters. **A** The brain microstructure is modeled as three compartments: free water (FW), intra-neurite, and extra-neurite spaces. The free water fraction (FWF), neurite density index (NDI), and orientation dispersion index (ODI) are derived from these compartments. **B** Representative axial slices from NODDI-derived maps for FWF, NDI, and ODI. Note: Figure adapted from Kraguljac et al. (2023), licensed under Creative Commons Attribution-NoDerivatives 4.0 International (CC BY-ND 4.0), available at http://mig.cs.ucl.ac.uk/index.php?n=Tutorial.NODDImatlab

NODDI decomposes the diffusion signal into three compartments: intracellular, extracellular, and free water compartments, allowing for the differentiation between tissue environments with distinct microstructural constituents (see Fig. 1). The intracellular compartment refers to the highly restricted space within neurites, bounded by the membranes of axons and dendrites (collectively known as neurites). This compartment provides the key metric—the neurite density index (NDI), which quantifies the proportion of space within a voxel occupied by neurites, thereby indexing neurite density—how tightly packed axons and dendrites are within the tissue (see Table 1). The free water compartment models the space occupied by cerebrospinal fluid (CSF), providing the metric known as free water volume fraction (FWF), which aims to estimate the extent of CSF contamination in the diffusion signal. Addressing partial volume effects from CSF is particularly important, as they can significantly bias diffusion measurements (Alexander et al. 2001; Vos et al. 2011). The extracellular compartment refers to the space surrounding the neurites, which is less restricted but still hindered by the presence of neurites. This space is occupied by glial cells and, additionally in gray matter, by neuronal cell bodies (somas). While the extracellular volume fraction is not directly estimated in NODDI, it can be inferred as the residual volume after accounting for the intracellular and CSF compartments.

**Table 1:** NODDI compartments, metrics, and key features

Compartment	Description	Key metrics	Microstructure assessed
Intracellular	Space within neurites (axons and dendrites), highly restricted by cell membranes	NDI ODI	NDI quantifies neurite density ODI captures neurite orientation variability, from coherent to dispersed arrangements
Extracellular	Space surrounding neurites, less restricted but influenced by neurites, glial cells, and neuronal somas (in gray matter)	Inferred residual volume	Reflects the remaining space after accounting for intracellular and CSF compartments
Free water	Space occupied by CSF with unrestricted diffusion	FWF	Estimates the extent of CSF contamination in diffusion signals

*NODDI* neurite orientation dispersion and density imaging, *NDI* neurite density index, *ODI* orientation dispersion index, *FWF* free water volume fraction, *CSF* cerebrospinal fluid

In addition to these compartmental volume fractions, NODDI provides a measure of the orientation dispersion index (ODI), which quantifies the variability in the orientation of neurites within a voxel, from highly coherent to more dispersed arrangements. For example, white matter structures with highly aligned axons, such as the corpus callosum, exhibit low ODI values, reflecting a dominant, single-direction alignment. In contrast, regions like the centrum semiovale, which contains bending and fanning axons, have moderate ODI values, indicating a less uniform but still organized orientation pattern. In the cerebral cortex and subcortical gray matter, in which dendritic processes sprawl in all directions, ODI values are typically higher, reflecting the complex, multi-directional orientation of neurites. This capacity to capture the varied orientation complexity of neurites makes ODI a valuable metric for studying both white and gray matter microstructures, particularly during the dynamic phases of brain development in infancy. A detailed technical review of the model’s underlying assumption and parameter estimation is beyond the scope of this review. Please refer to the original NODDI paper by Zhang et al. (2012), which outlines the theoretical framework and validation of the model.

Several software toolboxes have been developed to estimate NODDI model parameters. The original NODDI Matlab toolbox (Zhang et al. 2012) fits the NODDI model to multi-shell diffusion MRI data and generates voxel-wise maps of NDI, ODI, and FWF. Two widely used alternatives offer computational advantages: AMICO (Daducci et al. 2015) reformulates the model fitting as a linear system, dramatically reducing computation time with minimal accuracy loss, while CUDA diffusion modeling toolbox (cuDIMOT; Hernandez-Fernandez et al. 2019) uses GPU acceleration to speed up the nonlinear MRI model fitting process. These toolkits reduce computational demands and have made NODDI modeling practical for large-scale studies and facilitated broader adoption in the field.

### Biophysical basis and interpretation

NODDI, as a biophysical model, offers greater specificity by parametrizing the dMRI signal as a function of biologically meaningful parameters (e.g., axon density) in contrast to signal representation approaches, which provide summary statistics of the observed signal without making assumptions about the underlying tissue architecture (Novikov et al. 2018). NODDI, therefore, offers more specific and interpretable metrics for examining the microstructure of both white and gray matter. In white matter, NODDI aims to capture the density and organization of axons, which are long projections of neurons responsible for transmitting electrical signals across different parts of the brain. The NDI models axonal density, offering insights into the integrity and maturation of neural pathways. The ODI measures the variability in the fiber alignment, providing a view of how uniformly or diversely these fibers are oriented. Distinct from white matter, which is composed of long, coherently aligned fibers, gray matter primarily consists of neuronal cell bodies and branching dendrites. Dendrites are branch-like extensions of neurons that receive signals from other neurons, playing a key role in synaptic connections and neural communication (Spruston 2008). NDI in gray matter serves as a proxy for dendrite density, potentially indicating synaptic complexity and connectivity. Gray matter ODI may capture the multi-directional orientation of dendrites, which tend to branch out in all directions. NODDI in gray matter is therefore considered indicative of dendritic arborization complexity and the architectural layout of cortical and subcortical regions. Beyond neurons, there is emerging evidence that NODDI metrics may also reflect aspects of glial cells. For instance, Yi et al. (2019) observed that ODI might be sensitive to microglial density due to greater concentration in the extra-neurite space. Taken together, biologically meaningful parameters have made NODDI a valuable tool in various clinical and research settings, including studies of psychiatric disorders, neurodevelopment, and aging (Kamiya et al. 2020; Kraguljac et al. 2023).

NODDI-derived metrics (NDI and ODI) do not map one-to-one onto single biological processes (e.g., axonal growth, myelination, dendritic arborization, or synaptogenesis), which presents significant challenges for developmental interpretation. For instance, an increase in white matter NDI during infancy could result from increased axonal packing, axonal growth, or myelination—all of which restrict extracellular diffusion and raise the intracellular volume fraction (Jespersen et al. 2010). Similarly, changes in cortical NDI may reflect dendritic arborization, intracortical axonal growth, or glial proliferation (Yi et al. 2019). Researchers have adopted interpretive strategies that are grounded in neurodevelopmental timing and anatomical location. For example, Jelescu et al. (2014) interpreted increases in NDI within the corpus callosum during the first three years of life as likely reflecting active myelination, rather than an increase in axonal number. This interpretation is supported by evidence that the number of callosal axons is already near maximal at birth (Kostović and Jovanov-Milošević, 2006). Thus, it is important to interpret NODDI findings within a broader developmental framework and, when possible, support evidence from complementary modalities.

### Limitations and considerations

It is important to note that NODDI is not without limitations. The NODDI model, like all model-based approaches, relies on a host of assumptions about the underlying diffusion processes and tissue architecture. Specifically, the intracellular, extracellular, and free water compartments are modeled as a set of sticks (i.e., water primarily moving along the length of neurites), Gaussian anisotropic diffusion, and isotropic Gaussian diffusion (fixed at 3 μm^2^/ms diffusivity), respectively. Such compartmental assumptions, however, overlook several potentially important factors, including the inter-compartmental exchange of water molecules and non-Gaussian diffusion effects within the compartments (Jelescu et al. 2020). Taking a step back, even at a foundational level, such as the optimal number of compartments, consensus has not yet been reached (Kamiya et al. 2020). Additionally, to ensure stable parameter estimation, NODDI imposes several constraints. For example, NODDI assumes that the intracellular compartment diffusivity and the principal direction of the extracellular diffusion tensor are identical and fixed to an a priori value of 1.7 μm^2^/ms. This value was estimated from the corpus callosum (Szafer et al. 1995), and is assumed to be consistent across different brain regions and subjects (Alexander et al. 2010). However, this fixed diffusivity constraint may not be optimal across different populations (e.g., infants vs. adults) and tissue types (e.g., white and gray matter) (Guerrero et al. 2019). Diffusivity values in infants are generally lower than in adults, and using default adult-derived parameters (i.e., 1.7 μm^2^/ms) may bias microstructural estimates in pediatric populations. Similarly, optimal values may differ between white and gray matter (Guerrero et al. 2019). These diffusivity assumptions directly affect NDI and ODI estimates, making cross-study comparisons problematic when different parameter sets are used. Several considerations may help improve consistency, transparency, and interpretability in future work. First, clearly reporting diffusivity parameters and justifying their selection based on population characteristics would enhance reproducibility. Second, when resources permit, exploring the effects of varying diffusivity through sensitivity analyses or model fitting comparisons can provide insights into parameter robustness. Finally, when interpreting findings across studies, accounting for methodological differences and exercising caution in direct comparisons of absolute NDI or ODI values, unless modeling assumptions are matched, will strengthen conclusions.

Furthermore, the orientation distribution function is constrained to an axially symmetric Watson distribution, which models a single dominant orientation along with the spread of orientations around it, however this constraint limits the model’s flexibility in capturing complex tissue configurations. For more details about the rationale behind these model assumptions and parameter constraints, please refer to (Kraguljac et al. 2023; Zhang et al. 2012). Being aware of these assumptions allows researchers to recognize potential biases introduced by the model’s constraints, critically evaluate whether these constraints align with their study’s specific goals and populations, and facilitate more robust interpretations. This awareness also guides the development of strategies to validate findings. For example, researchers might relax or modify certain constraints, or combine NODDI with other imaging techniques to provide converging evidence and enhance the reliability of results (see Sect. “Future directions and summary”).

Despite these limitations, NODDI remains one of the few multicompartment models that have been extensively validated through histological studies, which are considered to be the gold standard for verifying imaging techniques. The ODI has been shown to correlate with histologically derived measures of neurite orientation dispersion in both animal and human studies (Grussu et al. 2017; Sato et al. 2017; Schilling et al. 2018), and the NDI has demonstrated correspondence with histologically measured neurite and axonal density in ex vivo tissue (Sepehrband et al. 2015). Additionally, a significant body of literature supports the repeatability and reproducibility of NODDI metrics. Scan–rescan studies have demonstrated excellent repeatability in the brain, with intra-subject coefficients of variation (CoV) for NDI and ODI generally below 5%, which is comparable to that of DTI (Andica et al. 2020; Chang et al. 2015; Chung et al. 2016; Granberg et al. 2017; Huber et al. 2019; Tariq et al. 2012). Thus, NODDI may be particularly well-suited for longitudinal studies, including early development, to capture the dynamic microstructural changes occurring in the brain. Nevertheless, inter-vendor reproducibility of NODDI appears slightly lower (CoV = 2.3–14%; Andica et al. 2020), and field strength is also an important consideration since it may have a significant effect on NODDI measures (Chung et al. 2016). Thus, data acquired from different sites or scanners should be interpreted carefully, and harmonization techniques are needed to ensure data consistency and interpretability (Fortin et al. 2017).

## Early microstructural development

Dynamic macrostructural and microstructural changes take place from the fetal stage through the first years after birth, representing one of the most dynamic periods in the human lifespan (Kostović et al. 2019). During gestation, the brain undergoes rapid and complex developmental processes, including neuronal proliferation, migration, aggregation, axonal growth, dendritic differentiation, and synapse formation. By the third trimester, essential structures such as the cortical plate, subplate, and thalamus are formed, with synaptogenesis already well underway (Kostović et al. 2019; Stiles and Jernigan 2010). All major long-distance fiber tracts, which begin forming as early as the 9th post-conception week, are observed by the end of the late preterm period (Vasung et al. 2017). Following birth, the brain continues its rapid growth and refinement. Brain volume, approximately 35% of its adult size by 2–3 weeks after birth (Gilmore et al. 2007), doubles within the first year and grows by an additional 15% during the second year, reaching roughly 80% of adult volume (Groeschel et al. 2010; Knickmeyer et al. 2008). This rapid expansion is primarily driven by increases in gray matter, which reflects changes in the neuropil, a network of dendrites, axons, and glial cells (Groeschel et al. 2010). Intensive dendritic and axonal arborization, along with spine growth, also occur during the first year (Mrzljak et al. 1991). Synaptogenesis surges at birth, with synaptic density steadily increasing throughout infancy (Glantz et al. 2007). Myelination, although initiated before birth, accelerates significantly in the neonatal period (Dubois et al. 2014; Paus et al. 2001). In early infancy, despite the weak myelination of white matter bundles, all major tracts can be observed via diffusion imaging and tractography, including commissural, projection, limbic, and associative bundles (Dubois et al. 2006; Kulikova et al. 2015), as well as short-range connections (Dubois et al. 2016b). By the end of the first postnatal year, useless or redundant axonal fibers are pruned, while ongoing myelination improves information transfer between distant brain regions and functionally essential connections. The number of oligodendrocytes and astrocytes in white matter increases drastically in the first three years of life, reaching approximately two-thirds of the adult count (Sigaard et al. 2016).

Given the rapid and complex changes occurring in the early brain, investigating brain development during this period is essential for understanding the foundational processes that support lifelong cognitive, social, and behavioral functions (Gilmore et al. 2018). Traditionally, brain development research has focused on macrostructural changes, such as overall brain volume. However, these gross measures lack the ability to capture cellular and microstructural processes, such as dendritic arborization, synaptogenesis, and myelination, and examining microstructural development can provide a more fine-grained understanding of the neural tissue that underpins macroscopic structural growth. The ability of diffusion imaging to non-invasively measure neuronal microstructure makes it an ideal tool to assess developmental changes in these features. NODDI, a biophysical model of diffusion MRI, can be a useful tool for mapping brain development with greater specificity, offering biologically meaningful metrics like NDI and ODI (see Table 2). By capturing microstructural architecture in both white and gray matter, NODDI enables a deeper understanding of the processes driving early brain development, helps identify sensitive periods, as well as sheds light on the potential origins of neurodevelopmental disorders.

**Table 2:** Early neurodevelopmental features and their potential reflection in NODDI metrics

Developmental feature	Trajectory in infancy	Possible NODDI correlate	Hypothetical interpretation
Axonal growth	↑	NDI ↑	Maturation of axons increase the intra-neurite volume fraction
Myelination	↑	NDI ↑; FWF ↓	Increased formation of compact myelin sheaths reduces extracellular water diffusion and increase the intra-neurite volume fraction
Dendritic arborization	↑	ODI ↑	Greater dendritic branching increases the angular dispersion of neurites, reflected in higher orientation dispersion index
Synaptogenesis	↑	NDI ↑; ODI ↑	Greater synaptic density may relate to a denser and more spatially complex microstructural environment, possibly increasing both the intra-neurite volume fraction and orientation dispersion

*NODDI* neurite orientation dispersion and density imaging, *NDI* neurite density index, *ODI* orientation dispersion index, *FWF* free water volume fraction

## Early brain microstructural development with NODDI

### White matter microstructural development in early life

**Fig. 2:**
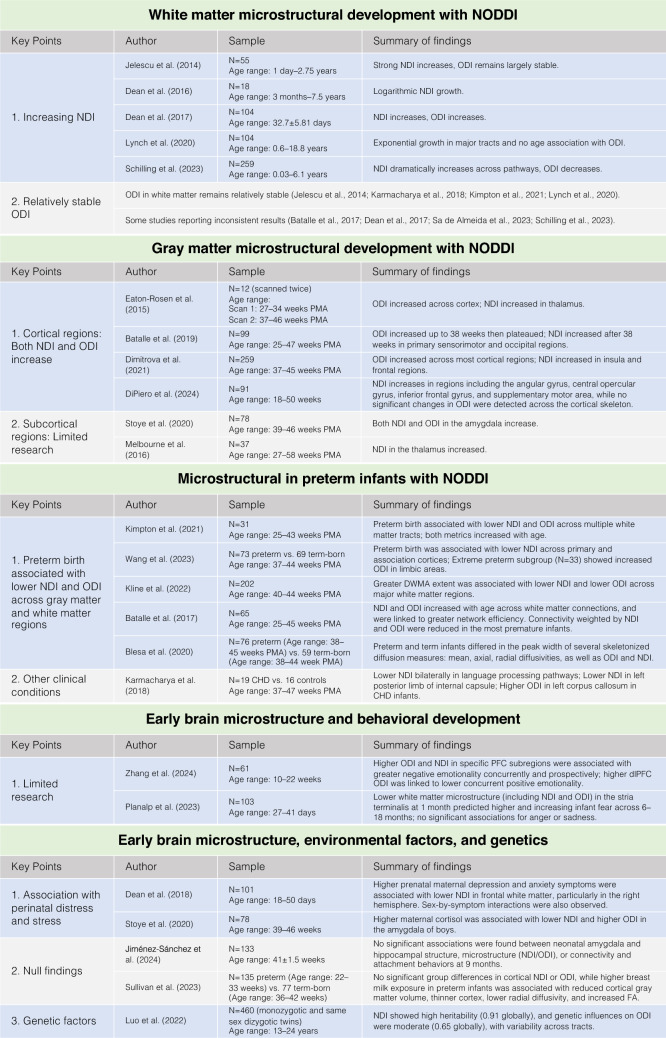
Summary of studies examining early brain microstructural development using NODDI. This figure summarizes key findings from studies investigating brain microstructure with NODDI in infancy. Each section highlights relevant central findings and sample characteristics (i.e., age, sample size)

NODDI has been used to investigate microstructural development from as early as the second trimester—drawing on data from premature infants—through the postnatal period (Batalle et al. 2017, 2019; Dean et al. 2016, 2017; Dimitrova et al. 2021; Eaton-Rosen et al. 2015; Fenchel et al. 2020; Jelescu et al. 2014; Kimpton et al. 2021; Kunz et al. 2014; Lynch et al. 2020; Melbourne et al. 2016; Schilling et al. 2023; Zhao et al. 2021; see Fig. 2. In a study of 13 newborns, Kunz et al. (2014) reported high NDI and low ODI values in early-maturing, tightly packed tracts (e.g., the corpus callosum and posterior limb of the internal capsule), and lower NDI values in later-maturing regions (e.g., the external capsule and periventricular crossroads of pathways). Regions with fiber crossings and fanning, such as the short association U-fibers and periventricular crossroads of pathways, exhibited the highest ODI values. These findings align with established white matter developmental patterns (Dubois et al. 2016a; Lebel and Deoni 2018) and demonstrate the utility of the NODDI model for characterizing early white matter microstructure and maturation during infancy.

In white matter, NDI shows a dramatic increase from birth, following a non-linear trajectory with the most rapid changes occurring early in life (Batalle et al. 2017; Dean et al. 2016, 2017; Jelescu et al. 2014; Karmacharya et al. 2018; Kimpton et al. 2021; Lynch et al. 2020; Schilling et al. 2023), while ODI in white matter generally remains stable (Jelescu et al. 2014; Karmacharya et al. 2018; Kimpton et al. 2021; Lynch et al. 2020), though some studies report inconsistent results (Batalle et al. 2017; Dean et al. 2017; Sa de Almeida et al. 2023; Schilling et al. 2023). Jelescu et al. (2014) examined WM microstructural changes over the first three years of healthy brain development (1 day to 2 years and 9 months) in 55 subjects and revealed a non-linear increase in intra-axonal water fraction (reflected in NDI metric) and in the tortuosity of the extra-axonal space (calculated as the ratio of axial to radial extracellular diffusivity) with age, particularly in the genu and splenium of the corpus callosum and the posterior limb of the internal capsule. ODI, by contrast, remains largely stable during this early developmental period. In another study of 18 typically developing children aged 3 months to 7.5 years, NDI exhibited a predominantly non-linear growth pattern, increasing logarithmically with age (Dean et al. 2016). Additionally, a study of 104 one-month-old infants found an increase in NDI across white matter regions, with ODI increases specifically in the right-hemisphere superior corona radiata, left-hemisphere cingulum, and posterior thalamic radiation. Both NDI and ODI demonstrated regional asymmetries across these white matter regions (Dean et al. 2017). More recently, a study with 104 subjects aged 0.6–18.8 years, observed exponential age-related growth in NDI across major white matter tracts, with rapid increases during infancy and early childhood followed by a plateau in adolescence, while ODI was not found to be significantly associated with age (Lynch et al. 2020). Furthermore, using data from the Baby Connectome Project (Howell et al. 2019), which included 259 subjects aged 0.03–6.1 years, Schilling et al. (2023) observed that while ODI decreases, NDI dramatically increases across 63 selected white matter pathways. NDI growth was particularly pronounced in anterior pathways compared to posterior pathways.

Overall, these studies reveal a rapid increase in NDI metrics in early life, likely driven primarily by myelination (Miller et al. 2012). The observed developmental trajectory across major white matter tracts aligns with established evidence that myelination progresses in an inferior-to-superior and posterior-to-anterior gradient, with primary sensory regions maturing before areas that support higher-order executive functions (Colby et al. 2012; Dubois et al. 2008; PI 1966). The increase in NDI is also consistent with findings from studies using other diffusion models, such as DTI (Dubois et al. 2016a; Ouyang et al. 2019), conducted over the same developmental period. Some inconsistency in ODI findings, however, may be attributed to differences in the age ranges examined and potential regional variations in developmental patterns. For instance, Schilling et al. (2023) analyzed four large public datasets spanning a wide age range (0–100 years), with only 259 subjects aged 0.03–6.1 years, which may lack the temporal resolution needed to capture the rapid, weekly, or monthly changes characteristic of early development, but rather reflects a relatively long-term change across the 0–6 year age range. In contrast, Dean et al. (2017) focused specifically on very early life (1 month of age ± 2 weeks) and observed an increase in ODI. Additionally, regional specificity may also play a significant role in ODI findings. For example, Jelescu et al. (2014) assessed changes in three highly coherent white matter tracts—the genu and splenium of the corpus callosum, as well as the posterior limb of the internal capsule—where they observed no ODI changes. There is evidence that, in humans, the number of axons in the corpus callosum is already at its maximum at birth (Kostović and Jovanov-Milošević, 2006), which may contribute to the limited changes observed. Therefore, more data is needed to highlight the importance of both finer temporal sampling and regional sampling in investigating early developmental trajectories. For example, the balance of myelination and axonal pruning may exert opposing effects on microstructure during this period, with myelination increasing microstructural organization and pruning potentially reducing it. Capturing these processes at a higher temporal resolution may help disentangle their relative contributions to microstructural changes in early development.

### Gray matter microstructural development in early life

In cortical gray matter, both NDI and ODI have been observed to increase rapidly from the late second trimester through the early postnatal period (Batalle et al. 2019; Dimitrova et al. 2021; DiPiero et al. 2024; Eaton-Rosen et al. 2015). Eaton-Rosen et al. (2015) analyzed data from two time points (soon after birth and at term-equivalent age) in 7 preterm infants born at or before 28 weeks, and found an increase in ODI across the cortex and an increase in NDI in the thalamus. In another study of 99 preterm infants scanned between 25 and 47 weeks postmenstrual age (PMA), Batalle et al. (2019) observed that ODI increased up to 38 weeks and then plateaued, while NDI began increasing only after 38 weeks. Regional analysis of cortical microstructure revealed that NDI increases after 38 weeks were primarily confined to primary motor and sensory regions. These findings suggest that cortical development between 25 and 38 weeks may predominantly reflect dendritic arborization and neurite growth, while development between 38 and 47 weeks may be dominated by increases in cellular and organelle density. Using data from the developing Human Connectome Project (dHCP), Dimitrova et al. (2021) analyzed 259 healthy term-born infants scanned between 37 and 45 weeks PMA and found that older PMA at scan was associated with higher ODI across the brain, with the steepest increases in the parietal and temporal lobes, but not in the somatosensory cortex. Older age was additionally associated with higher NDI in the insula and frontal lobe. In contrast, preterm infants (n = 76) in dHCP data scanned at term-equivalent age, exhibited lower NDI across the posterior cortex compared to term-born infants. A recent study by DiPiero et al. (2024) scanned a sample of 91 infants at approximately 1 month of age and observed widespread increases in cortical NDI across regions including the angular gyrus, central opercular gyrus, inferior frontal gyrus, and supplementary motor area, while no significant changes in ODI were detected across the cortical skeleton.

Overall, these limited studies provide valuable insights into the microstructural changes taking place in cortical regions during the perinatal period, highlighting a dramatic increase in dendritic arborization and synapse production after birth (Huttenlocher 1990; Huttenlocher and Dabholkar 1997), which is likely driving microstructural changes in the cortex. During the perinatal stage, the radial alignment of cortical neurites becomes less pronounced as dendritic trees grow and synaptic connections form, resulting in increasingly complex microstructural geometries that likely contribute to the observed increase in ODI. The increase in NDI observed in infants may reflect both the expansion of neurite length and branching as well as a rise in the density of cellular components, such as organelles. However, much of the current work relies heavily on preterm populations, which may exhibit developmental trajectories that differ from those of typically developing infants (López-Guerrero and Alcauter 2023; Padilla et al. 2015; Smyser et al. 2010). Moreover, existing data predominantly covers the perinatal period, leaving the early infancy period largely unexplored. Such work is essential for understanding microstructural development, sensitive periods, and early neural plasticity, as well as linking observed macrostructural growth to underlying microstructural processes. For example, it remains unclear how cortical microstructural maturation processes, such as synaptogenesis and dendritic arborization, support the development of early cognitive functions like attention and language, and how disruptions in microstructural properties may impact higher-order cognitive and emotional functioning later in life.

To date, microstructural development in gray matter subcortical regions has not been extensively examined in infants. A few studies have reported increases in thalamic NDI in very preterm samples, including 37 infants (Melbourne et al. 2016) and 7 infants (Eaton-Rosen et al. 2015). In a separate study of 78 newborns, both NDI and ODI in the amygdala were positively associated with gestational age at birth and age at scan (Stoye et al. 2020). Similar investigations in adolescents have provided additional insights. In a sample of 61 adolescents aged 8–22 years, Azad et al. (2021) found significant age-related increases in NDI within amygdala subnuclei (i.e., the lateral nucleus, dorsal and intermediate divisions of the basolateral nucleus, ventral division of the basolateral nucleus, and paralaminar nucleus). Additionally, age-related NDI increases were observed in amygdala-associated white matter pathways (i.e., the anterior commissure, ventral amygdalofugal pathway, cingulum, and uncinate fasciculus). It would be of interest to extend this line of research to early life—a period with rapid growth in subcortical regions. For example, the amygdala undergoes significant development during prenatal and early postnatal life (Mulc et al. 2024; Uematsu et al. 2012) and plays a central role in emotional processing and social responses (Janak and Tye 2015; Phelps and LeDoux 2005). Examining its microstructural development in vivo during early life may provide critical insights into how its early neuronal and synaptic maturation shapes later emotional functioning (Pecheva et al. 2024).

### Cortical–white matter interactions

Interestingly, emerging evidence suggests that the development of cortical NDI might be closely interlinked with white matter maturation. Liu et al. (2022) investigated spatiotemporal developmental patterns of five association fibers (indexed by metrics from DTI and advanced fiber-specific diffusion analysis) and their connected cortical regions (indexed by NDI and ODI) in 108 healthy preterm-born infants aged 39.9 to 59.9 weeks. They found that both the association fibers and connected cortical gray matter demonstrated faster development in anterior regions compared to posterior regions. Moreover, they demonstrated that the observed developmental patterns were not biased by prematurity by showing similar patterns in a matched sample of 31 term- and preterm-born neonates from the dHCP dataset. Mediation analysis further revealed mutual mediation between the development of association fibers and connected cortical NDI, indicating a neuron-oligodendroglia interaction (Thornton and Hughes 2020). Furthermore, the mediation analysis indicated a stronger mediation effect of cortical NDI on white matter development than vice versa, highlighting a potentially dominant impact of neuronal activity on oligodendroglia in facilitating axonal growth and neural circuit formation.

## Microstructural alterations in preterm infants

NODDI has also been applied to study brain microstructure in preterm infants, providing insights into how premature birth may affect gray and white matter maturation (Batalle et al. 2017; Blesa et al. 2020; Dimitrova et al. 2021; Kelly et al. 2016; Kimpton et al. 2021; Kline et al. 2022; Sa de Almeida et al. 2023; Sullivan et al. 2020; Vaher et al. 2022; Wang et al. 2023). In a study of 31 preterm neonates born between 24 and 36 weeks gestational age (GA) and scanned between 25 and 43 weeks PMA, all of whom demonstrated normal neurodevelopmental outcomes at 2 years, higher PMA was associated with increased NDI in the cingulum, corticospinal tract (CST), and fornix among the five selected tracts (cingulum, fornix, CST, optic radiations, and inferior longitudinal fasciculus) (Kimpton et al. 2021). No significant associations were observed between PMA and ODI. Additionally, GA and GA-by-PMA interactions showed significant effects on NDI, with higher GA linked to a more rapid increase in NDI compared to lower GA. However, the negative association between GA and NDI appears counterintuitive, given the observed increase in NDI with PMA. Sex and sex-by-PMA interactions were also associated with NDI. Note that these findings should be interpreted with caution due to the study’s small sample size. More recently, a study compared 73 preterm infants scanned at term-equivalent age (37–44 weeks) with 69 term-born infants and found lower NDI in both primary and higher-order association cortices in preterm infants compared to term-born infants (Wang et al. 2023). Additionally, the extreme preterm subgroup (n = 33) showed increased ODI in regions such as the orbitofrontal cortex, fronto-insular cortex, entorhinal cortex, posterior cingulate gyrus, and medial parieto-occipital cortex. In another study of 202 infants born preterm at ≤ 32 weeks gestational age, diffuse white matter abnormalities (DWMA) were identified in regions exhibiting hyperintense signals on T2-weighted imaging (Kline et al. 2022). White matter areas marked by DWMA showed lower ODI and NDI values, indicating potential alterations in microstructural development. Additionally, NDI correlated with the extent of DWMA across all major white matter regions except the cerebellum, while ODI demonstrated a significant correlation with DWMA extent specifically in the centrum semiovale, corona radiata, and temporal lobe, providing valuable insights into the biological underpinnings of DWMA in preterm infants. Blesa et al. (2020) used a histogram-based method to analyze the peak width of skeletonized diffusion MRI measures—a metric defined as the difference between the 95th and 5th percentiles of voxel values within the white matter skeleton—in 135 neonates (76 preterm, 59 term-born) at term-equivalent age. They found that preterm and term infants differed in the peak width of several skeletonized diffusion measures: mean, axial, and radial diffusivities, as well as ODI and NDI. Among these measures, the peak width of skeletonized NDI was most effective at distinguishing between preterm and term infants, achieving 81 ± 10% classification accuracy.

The white matter connectivity of preterm infants has also been examined at the network level. Using a robust graph theory analysis approach, Batalle et al. (2017) studied 65 neonates born between 24 and 41 weeks GA and scanned between 25 and 45 weeks PMA and found that both NDI and ODI increased across white matter connections with age, and higher NDI and ODI correlated with stronger global and regional efficiency. Notably, connectivity weighted by both NDI and ODI showed significant reductions in the most premature infants, particularly in connections associated with higher-order cognitive and socio-emotional functions. Additionally, NDI in core connectivity—key structural connections within the brain—showed little change in preterm infants, whereas NDI in peripheral connections was significantly lower compared to term-born healthy controls. This suggests that the preterm brain may demonstrate resilience to developmental disruptions in core connections, while peripheral connections, often linked to important aspects of cognition and behavior, appear more vulnerable following preterm birth.

NODDI has also been used to assess the effects of intervention on brain microstructural development in preterm infants. In a study of 40 preterm infants born between 24 and 32 weeks GA, infants were randomized into two groups: 21 received a daily music intervention, while 19 served as controls (Sa de Almeida et al. 2023). NODDI metrics were assessed both before the intervention (at 33 weeks GA) and again at term-equivalent age. In both groups, ODI significantly increased over time across all evaluated cortical regions, reflecting ongoing maturation. However, the music intervention group showed a significantly greater increase in ODI in cortical paralimbic regions—specifically the insulo-orbito-temporopolar complex, precuneus/posterior cingulate gyrus, and the auditory association cortex—areas critical for auditory, cognitive, and socio-emotional processing. These findings suggest that music interventions may support enhanced intracortical multidirectional complexity in preterm infants.

Beyond studies of preterm infants, NODDI has also been applied to examine neonates with congenital heart disease (CHD). Karmacharya et al. (2018) investigated 19 neonates with CHD, aged 37 to 41 weeks and found lower NDI bilaterally in pathways essential for language processing, including the uncinate fasciculus, corpus callosum (CC), superior fronto-occipital fasciculus, and the left posterior limb of the internal capsule. Additionally, ODI was found to be higher in the left CC in infants with CHD compared to a control group of typically developing neonates (N = 16), aged 38 to 47 weeks. These findings suggest that CHD may be associated with alterations in the development of white matter pathways important for language and cognitive processing.

In sum, preterm birth has been associated with lower NDI and ODI across cortical gray matter and white matter regions, with considerable variability in individual deviations from typical development among preterm infants. This suggests that the abrupt transition to an extrauterine environment through preterm birth may disrupt cortical microstructure and growth. Indeed, the dynamic and complex cellular events occurring during the last trimester make the developing cortex particularly vulnerable to such perturbations (Volpe 2019). Although the precise mechanisms—whether compensatory or pathological—underlying these microstructural changes in the preterm brain remain largely unknown, they likely involve a disruption in dendrite and spine formation, a general reduction in the morphological complexity of cortical neurons, and alterations in axonal growth and myelination patterns in white matter (J. M. Dean et al. 2013).

## Early brain microstructure, behavioral development, environmental factors, and genetics

### Early brain microstructure and behavioral development

To date, only a limited number of studies have explored the associations between NODDI microstructural metrics and early sensorimotor, cognitive, and socioemotional development in early infancy (Planalp et al. 2023; Zhang et al. 2024). For example, white matter microstructure at 1 month, particularly in the stria terminalis and sagittal stratum, was found to predict increases in fear—but not sadness or anger—across infancy (Planalp et al. 2023). Recently, a study investigated how microstructural markers within prefrontal cortical subregions relate to infant negative emotionality and positive emotionality, both concurrently and prospectively. Specifically, at 3 months, greater ODI in the rostral anterior cingulate cortex and greater NDI in the caudal anterior cingulate cortex (ACC) were associated with higher concurrent negative emotionality (n = 61); greater ODI in the lateral orbitofrontal cortex was associated with higher prospective negative emotionality at 9 months (n = 50); and greater ODI in the dorsolateral prefrontal cortex was associated with lower concurrent positive emotionality (Zhang et al. 2024). This research represents an important step toward understanding how early brain microstructure might shape fundamental aspects of emotional and behavioral development in infancy, shedding light on the neural basis of developing emotional regulation. The potential to use microstructural markers in infancy to identify or complement early indicators of future psychopathology risk is notable, warranting further efforts in this line of research to advance our understanding of the neural basis of early development and to inform interventions that support optimal health from the earliest stages.

### Early brain microstructure, environmental factors, and genetics

Environmental factors play a crucial role in shaping early brain development, influencing both structural and functional neural outcomes (Gao et al. 2019; Miguel et al. 2019). Recent research has increasingly highlighted associations between various environmental exposures—such as prenatal stress and maternal distress—and early brain microstructure (Dean et al. 2018; Stoye et al. 2020). In a study of 101 mother–infant dyads, higher levels of prenatal depression and anxiety symptoms were associated with lower NDI in the right frontal white matter of infants at one month of age (Dean et al. 2018). Additionally, significant maternal symptom-by-sex interactions were observed for both NDI and ODI in the sagittal stratum, posterior thalamic radiations, and white matter adjacent to the hippocampus—higher prenatal maternal symptoms were linked to increased NDI and ODI in boys but decreased NDI and ODI in girls. Another study of 78 mother–newborn dyads measured maternal hair cortisol concentration (HCC) shortly after delivery and found that both NDI and ODI in the amygdala were positively associated with gestational age at birth and age at scan. Higher maternal HCC was linked to lower amygdala ODI in girls compared to boys. When analyzed separately by sex, higher maternal HCC was associated with lower amygdala NDI and higher amygdala ODI in boys (Stoye et al. 2020). These data suggest that maternal distress symptoms and perinatal stress might be associated with early microstructural development in a sexually dimorphic manner, as well as highlight the importance of the perinatal period for shaping early neural architecture.

However, null findings have also been reported. With 133 preterm infants born at 32 weeks GA, Jiménez-Sánchez et al. (2024) found no significant associations between neonatal amygdala or hippocampal microstructure (indexed by NDI and ODI) or structural connectivity (indexed by whole-brain connectivity NDI and ODI, seeding from amygdala and hippocampus) and infant attachment behaviors assessed at nine months of age using the Still-Face Paradigm. Sullivan et al. (2020) compared three groups: 67 preterm infants with high breast milk exposure, 68 with low exposure, and 77 term-born controls. They found no statistically significant differences in cortical NDI or ODI between groups. However, among preterm infants, those with higher breast milk exposure showed cortical features more similar to term-born infants: smaller cortical gray matter volume, thinner cortex, lower radial diffusivity, and higher FA.

Further, there is evidence that genetic factors may play a key role in the development of white matter microstructure as assessed by NODDI (Luo et al. 2022). In a cohort of monozygotic and same-sex dizygotic twins (N = 460) aged 13 to 24 years, Luo et al. (2022) estimated the relative contributions of additive genetic factors, common environmental influences, and unique environmental factors to NODDI measures. Genetic factors accounted for 91% of the variation in global NDI and 65% in global ODI, with NDI demonstrating particularly high heritability across 30 selected tracts. These findings highlight the significant genetic influence on white matter microstructure.

## The relevance of NODDI to developmental neuroscience

NODDI offers a valuable approach for assessing microstructural changes as they unfold, including how variability in microstructural development maps onto functional and behavioral differences among infants. For instance, by integrating NODDI-derived microstructural indices with functional MRI, researchers can ask how microstructural properties support or interact with functional brain activity and connectivity, advancing our understanding of how neural architecture and functional dynamics reciprocally shape neurodevelopment. For example, Teillac et al. (2017) found colocalization of neurite density and functional activation in the primary motor cortex, language network, and visual cortex, highlighting a potential link between neurite density and local signal processing that has not been consistently observed in macrostructural features such as cortical thickness and surface area (Evangelista et al. 2021). Moreover, early neural development lays the foundation for emerging motor, cognitive, and socioemotional abilities (Cruz et al. 2022; Hadders-Algra 2018), and NODDI can provide a microstructural perspective on how fundamental neurodevelopmental processes may shape these emerging behaviors. For example, a study, examining adult participants learning a complex dynamic balancing task over four weeks, found increased ODI in key motor-related regions, including the primary sensorimotor, prefrontal, premotor, and cingulate motor areas, during the training period but not during the control period (Lehmann et al. 2023). Importantly, the degree of ODI change correlated with performance improvements and was independent of macrostructural alterations in tissue density, cortical thickness, and intracortical myelin. These findings suggest that structural modulation of neurites may be a key mechanism supporting complex motor learning, and its application to infant studies may help clarify neural mechanisms underlying the acquisition of foundational motor skills and other emerging abilities.

Infants exhibit remarkable individual differences in development (Rothbart and Derryberry 2013). For instance, some infants demonstrate greater sensitivity to stimuli and stronger affective responses than others. Developmental neuroscientists have long been intrigued by the neural mechanisms underlying these variations, as understanding the neurobiological basis of early individual differences provides key insights into the origins of later cognitive, emotional, and behavioral outcomes. For gray matter, high-resolution T1- and T2-weighted MRI has long been the method of choice for investigating cortical and subcortical structures. However, in recent years, diffusion MRI has shown increasing promise for its unique insights into the cellular components of the cortex (Assaf 2019). Evidence from studies on aging and neurodegenerative diseases suggests that tissue properties derived from T1-weighted imaging (e.g., gray matter volume and cortical thickness) exhibit limited covariation with NODDI-derived microstructural metrics (Bai et al. 2022; Mak et al. 2021; Nazeri et al. 2015), and diffusion imaging indices may be sensitive to detecting aspects of neuroplasticity in gray matter that extend beyond conventional imaging methods (Assaf 2019; Nazeri et al. 2020; Tavor et al. 2013; Vukovic et al. 2021). NODDI thus offers an additional layer of information that complements existing approaches for studying variability in early neural development. Yet, as previously reviewed, its application in infancy research remains limited. For example, mental health poses a major public health concern (Patel et al. 2007), and functional dysregulation as well as morphological alterations in neural systems involved in arousal and salience detection—such as the amygdala and anterior cingulate cortex—have been implicated in various forms of psychopathology (Monk 2008). However, it remains unclear whether these patterns emerge at the microstructural level in early infancy, which can help address questions such as whether a neural basis for later-emerging psychopathology exists at this stage, and provides a more integrated understanding of neurodevelopment across multiple levels, bridging microstructural properties with functional organization and behavioral outcomes.

Furthermore, NODDI may serve as a useful tool for understanding atypical development (Nazeri et al. 2020), including detecting in vivo microstructural differences associated with neurodevelopmental disorders, such as autism spectrum disorder (ASD). Recently, NODDI has been used to investigate microstructure in adults with and without ASD, and found lower NDI in white matter tracts (Andica et al. 2021) and cortical regions (Arai et al. 2023; DiPiero et al. 2023a ) in the ASD group. Furthermore, ASD is associated with relatively consistent microstructural findings in the prefrontal cortex, hippocampus, and cingulate cortex, which likely reflect region-specific abnormalities in neuronal morphology and cytoarchitectural organization (Varghese et al. 2017). ASD emerges early in development, during a critical period for dendritic growth, and disruptions to these processes—particularly around age three—are hypothesized to increase ASD risk (Hutsler and Zhang 2010). This aligns with findings from postmortem and animal model studies that report alterations in dendritic spine density in ASD (Copf 2016; Martínez-Cerdeño, 2017). Compared to histological studies, which provide valuable insights into cellular-level pathology but are limited in their ability to track neural development over time, in vivo imaging offers a powerful alternative for longitudinal investigation of tissue microstructure in neurodevelopmental disorders. By enabling researchers to examine how brain microstructure evolves across different developmental stages, NODDI has the potential to advance understanding of disorder progression. Moreover, neurodevelopmental disorders often follow a gradual trajectory, with behavioral symptoms manifesting later after underlying neural differences have already taken shape. Assessment of underlying histopathological abnormalities in these disorders, which can potentially manifest earlier in the disease process, is more likely to provide a crucial window for focal targets for treatments.

Other than being useful in studying typical and atypical neurodevelopment, dMRI and NODDI also have the potential to advance our understanding of the associations between early-life experiences and brain development. Neurodevelopment is shaped by a complex interplay of genetic and environmental factors, with early-life experiences playing a crucial role in influencing brain structure and function. Developmental plasticity conceptualizes brain development as a dynamic, adaptive process shaped by reciprocal interactions between neural architecture, environmental context, genetics, and behavior—each factor continuously influencing and reshaping the others over time (Gottlieb 1991; Johnson 2001). For example, early environmental exposures may influence genetic expression through mechanisms such as epigenetics (Meaney 2010; Moore 2015), which in turn could alter neural pathways, ultimately shaping behaviors that feedback into the environment. This interactive process highlights that brain development is not a one-way path but rather a multidirectional, evolving system in which each factor exerts constant, adaptive influence on the others (Karmiloff-Smith 2006). In early life, the brain undergoes rapid growth, with significant microstructural development supporting emerging neural circuits and functional networks. Small deviations during these critical periods can have cascading effects on the neural system, leading to alterations across both basic and higher-order cognitive domains over time (Johnson 2001; Karmiloff-Smith 2009). As reviewed in the previous section, recent research has demonstrated associations between environmental exposures—such as prenatal stress and maternal distress—and early brain microstructure, as reflected in NODDI metrics (D. C. Dean et al. 2018; Stoye et al. 2020). Additionally, preterm birth has been linked to lower NDI and ODI across cortical gray matter and white matter regions, likely reflecting disrupted dendritic and spine formation, reduced morphological complexity of cortical neurons, and alterations in axonal growth and myelination patterns in white matter (J. M. Dean et al. 2013; Volpe 2019). NODDI has already shown promise in providing an additional layer of understanding regarding how environmental factors shape early brain development, although its application in this area remains limited.

## Applications of NODDI in infant neuroimaging analyses

NODDI-derived metrics can be analyzed using a range of strategies depending on the research question and scale of interest. In infant neuroimaging, these approaches fall into three main categories: voxelwise, region-of-interest (ROI), and tractography-based analyses. Each method offers unique advantages for examining early microstructural development and has been used to study group differences, developmental trajectories, and brain–behavior associations during infancy.

Voxelwise analyses examine metrics at individual voxels across the brain, enabling whole-brain investigations without requiring predefined regions. Tract-based spatial statistics (TBSS; Smith et al. 2006) is a common approach that projects individual diffusion maps onto a mean white matter skeleton for voxel-level statistical comparisons. For example, Kelly et al. (2016) used TBSS to compare white matter NODDI metrics in seven-year-old children born very preterm (N = 145) versus full-term (N = 33), finding higher ODI in the preterm group across major white matter tracts and significant associations between both NDI and ODI and neurodevelopmental outcomes. Voxelwise analysis can also be applied to cortical gray matter using gray matter-based spatial statistics (GBSS; Nazeri et al. 2015, 2017). DiPiero et al. (2024) refined the original GBSS framework, leveraging NODDI-based ODI maps, to enhance cortical delineation in the infant brain, revealing widespread increases in cortical NDI within sensorimotor and language-related regions over the first month of life.

ROI-based analyses extract NODDI metrics from predefined regions and compare averages between groups or correlate them with behavioral outcomes. This approach is straightforward and well-suited for hypothesis-driven questions. For example, Zhang et al. (2024) examined whether prefrontal cortex microstructure at age one month was associated with early emotionality. A key methodological consideration involves how voxel values are averaged within ROIs. The conventional approach treats all voxels equally when calculating ROI means, but this can introduce bias, especially in regions with CSF partial volume effects. Parker et al. (2021) developed a tissue-weighted mean that addresses this problem by using NODDI-derived tissue fraction to weight each voxel’s contribution based on its tissue content. This method provides more accurate representation of underlying tissue microstructure and reduces bias in ROI estimates and group comparisons, particularly when ROIs include CSF-contaminated voxels. Using such correction strategies is therefore important in NODDI studies to mitigate partial volume effects and improve the reliability of ROI-level findings.

Tractography-based analyses investigate microstructural development within specific white matter pathways or across structural networks. One approach involves averages NODDI values across the entire tracts to assess tract-level microstructure. Along-tract analysis samples NODDI values at multiple points along a tract’s trajectory, revealing spatial variations in white matter maturation within the same tract (Samuel Groeschel et al. 2014). Kimpton et al. (2021) used this approach in term-born neonates, finding asynchronous white matter maturation both within and between tracts—for example, the cingulum showed high NDI and low ODI at its center, while the fornix showed peak NDI and lowest ODI at anterior and posterior segments. Tractography can also be extended to network-level investigations where structural connections are weighted by NODDI metrics. Batalle et al. (2017) demonstrated that connectivity weighted by NDI and ODI was lower among the most premature infants, particularly in connections supporting higher-order cognitive and socio-emotional functions.

These analytic approaches demonstrate NODDI’s versatility in infant neuroimaging research, enabling investigation of early brain microstructure from focal regional differences to whole-brain networks.

## Future directions and summary

The vast majority of NODDI-based research in infants to date has been cross-sectional with limited sample sizes and restricted temporal coverage. Although these studies have provided meaningful insights, NODDI remains relatively underutilized during this critical period of rapid brain development, presenting the potential for advancing our understanding of early neurodevelopment (see Box 1). With growing interest in NODDI in developmental neuroscience, a critical next step is to revisit and evaluate its modeling assumptions in the context of infancy. Key aspects—such as the number of free parameters, the mathematical links between model components, and the values of fixed diffusivity—may not be optimal for the microstructural environment of the infant brain. Refining or adapting these parameters could improve model accuracy and interpretability during this early stage of neurodevelopment.

In addition to refining the model, another area for future research is to examine how NODDI metrics complement traditional T1-weighted based measurements of gray matter structure (e.g., thickness, area, volume, curvature, gyrification). While previous studies in adults and neurodegenerative disorders have demonstrated NODDI’s capacity to provide additional microstructural information (Bai et al. 2022; Mak et al. 2021; Nazeri et al. 2015; Vogt et al. 2020), direct quantification of the relations between NODDI and conventional structural metrics in early development is needed. For example, do regions with greater cortical thickness consistently show higher neurite density? Does orientation dispersion correlate with local gyrification patterns? Such questions can help clarify the unique information NODDI provides, its correlation with existing T1-weighted measures, and whether it offers complementary or redundant insights. As a relatively new modeling technique, establishing these relations could help validate NODDI metrics against well-established structural measures, and understanding the degree of overlap and unique contributions of NODDI metrics will be helpful for understanding its added value in developmental research and improving its application for studying early brain maturation.

Combining the NODDI model with other diffusion MRI modeling techniques or even integrating other imaging modalities may yield novel microstructural measures that help enhance our understanding of early brain development. One example is the MRI g-ratio framework (Stikov et al. 2015), which combines NODDI with myelin-sensitive techniques to estimate the ratio of inner-to-outer axon diameter (g-ratio), an index of myelination. Dean et al. (2016) estimated g-ratio by combining mcDESPOT (multi-component driven equilibrium single pulse observation of T1 and T2) and NODDI, and revealed a logarithmic decrease in g-ratio indices in 18 typically developing children aged 3 months to 7.5 years,indicating a gradual shift toward mature myelination patterns with age.

Additionally, integrating NODDI with other imaging modalities or biologically-informed data that capture distinct tissue properties facilitates leveraging complementary information to infer underlying neurodevelopmental processes. For example, Nazeri et al. (2022) revealed heterogeneous, age-associated growth patterns of white matter maturation during the early postnatal period by applying multivariate pattern analysis to T2w/T1w signal ratio maps from the dHCP (n = 342 newborns, scanned at 35–45 weeks PMA), findings that were subsequently replicated in another independent developmental cohort (eLABE, n = 239, scanned at 38–44 weeks PMA). The majority of T2w/T1w signal variations could be accounted for by NODDI microstructural indices (NDI, ODI, and free water content; R^2^ = 0.50–0.85) and were further associated with histological properties, indicating that white matter maturation likely reflects underlying microstructural and histological features. This study exemplifies an integrative approach, combining complementary imaging methods across large cohorts and leveraging overlapping and complementary insights, to deliver a more comprehensive and nuanced understanding of early brain development.

Diffusion MRI, as a non-invasive imaging method, has become a useful approach for probing brain microstructure. dMRI allows for estimating micron-scale displacement of water molecules, enabling detailed mapping of the brain’s cellular environment and capturing finer features of tissue architecture. Recently, NODDI, as a biophysical model of diffusion data, has started to be applied in studies of early brain microstructure in infancy. By providing biologically interpretable metrics, NODDI offers promising insights into the cellular architecture and microstructural processes across both white and gray matter. Its utility in early life also extends to uncovering associations between brain microstructure and environmental exposures, genetic factors, and emerging behavioral functioning, highlighting NODDI’s potential to deepen our understanding of neurodevelopmental contexts and trajectories. However, the use of NODDI in infant imaging remains limited, with relatively few studies conducted to date. In particular, mapping specific microstructural characteristics to behavioral development is still in its early stages, with considerable potential to advance our understanding of the neural basis of sensorimotor, cognitive, and socioemotional outcomes, as well as neurodevelopmental divergence.

**Figure:**
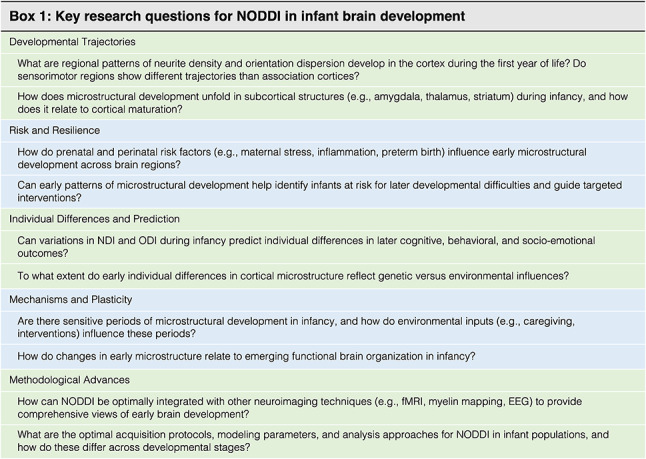


## Data Availability

No datasets were generated or analysed during the current study.
